# Concentration-Dependent Inhibitory Effect of Baicalin on the Plasma Protein Binding and Metabolism of Chlorzoxazone, a CYP2E1 Probe Substrate, in Rats In Vitro and In Vivo

**DOI:** 10.1371/journal.pone.0053038

**Published:** 2013-01-02

**Authors:** Na Gao, Dan Zou, Hai-Ling Qiao

**Affiliations:** 1 Department of Clinical Pharmacology, School of Medicine, Zhengzhou University, Zhengzhou, P. R. China; 2 Department of Histology and Embryology, Henan Medical College for Staff and Workers, Zhengzhou, P. R. China; Concordia University Wisconsin, United States of America

## Abstract

Some of the components found in herbs may be inhibitors or inducers of cytochrome P450 enzymes, which may therefore result in undesired herb-drug interactions. As a component extracted from *Radix Scutellariae*, the direct effect of baicalin on cytochrome P450 has not been investigated sufficiently. In this study, we investigated concentration-dependent inhibitory effect of baicalin on the plasma protein binding and metabolism of chlorzoxazone (CZN), a model CYP2E1 probe substrate, in rats in vitro and in vivo. Animal experiment was a randomized, three-period crossover design. Significant changes in pharmacokinetic parameters of CZN such as C_max_, t_1/2_ and V_d_ were observed after treatment with baicalin in vivo (*P*<0.05). C_max_ decreased by 25% and 33%, whereas t_1/2_ increased by 34% and 53%, V_d_ increased by 37% and 50% in 225 mg/kg and 450 mg/kg baicalin-treated rats, respectively. The AUC and CL of CZN were not affected (*P*>0.05). Correlation analysis showed that the changes in CZN concentrations and baicalin concentrations were in good correlation (r>0.99). In vitro experiments, baicalin decreased the formation of 6-OH-chlorzoxazone in a concentration-dependent manner and exhibited a competitive inhibition in rat liver microsomes, with a Ki value of 145.8 µM. The values of C_max_/Ki were 20 and 39 after treatment with baicalin (225 and 450 mg/kg), respectively. Protein binding experiments in vivo showed that the plasma free-fraction (fu) of CZN increased 2.6-fold immediately after baicalin treatment (450 mg/kg) and in vitro showed that baicalin (125–2500 mg/L) increased the unbound CZN from 1.63% to 3.58%. The results indicate that pharmacokinetic changes in CZN are induced by inhibitory effect of baicalin on the plasma protein binding of CZN and CYP2E1 activity.

## Introduction

Baicalin is a major flavone extracted from *Radix Scutellariae*, a plant which was widely used in traditional Chinese herbal medicine [1], [2]. Baicalin has been reported to possess a wide variety of pharmacological properties including anti-inflammatory, anti-oxidant, anti-viral, anti-cancer properties,and scavenging potential [3]. As a main component, baicalin has been used in a variety of preparations such as Huang-Lian-Jie-Du-Tang, San-Huang-Xie-Xin-Tang, Da-Chai-Hu-Tang, and Xiao-Chai-Hu-Tang et al [4]. These herbal medicines have been used in Asia since ancient times and have also been taken by European and American as remedy supplements and herbal teas in recent years [5], [6].

Some of the components found in herbs may be inhibitors, inducers, or substrates of cytochrome P450 (CYP) enzymes, and may cause undesired herb-drug interactions, and potentially limit its clinic application. CYP enzymes are heme-thiolate proteins that are responsible for the oxidative metabolism of numerous xenobiotics as well as endogenous substrates. The activities of CYP enzymes can be increased or decreased by many drugs, which is the main reason for drug-induced toxicity via drug–drug interactions [7]. It had demonstrated that *Radix Scutellariae* and its extracts, including baicalin, influenced the pharmacokinetics of co-administered cyclosporine [8]. Previous studies have found that baicalin enhanced the levels of liver microsomal CYP and selectively induced CYP1A1, 2B1, and 2C11 in mice [9]. Jang et al reported that oral treatment to mice with baicalin resulted in a significant decrease in acetaminophen-induced CYP2E1 activity together with its inhibition of acetaminophen-induced CYP2E1 expression [10]. Recent studies had found that baicalin could significantly induce CYP2B6-catalyzed bupropion hydroxylation and had no effect on gene expression of CYP3A4 and MDR1 [4], [11]. In summary, the effect of baicalin to different kinds of cytochrome P450 has not been completely understood and the direct relationship between baicalin and CYP2E1 is still unclear.

CYP2E1 is a natural ethanol-inducible enzyme and responsible for six percent drug metabolism involving a diversity of drugs, including alcohols, monocyclic compounds (e.g., benzene, p-nitrophenol), bicyclic heterocycles (e.g., coumarin,) and even fatty acids [12].The probes for CYP2E1 activity usually are chlorzoxazone (CZN), p-nitrophenol, aniline, and N-nitrosodimethylamine. Among these, CZN, a centrally-acting agent for painful musculoskeletal conditions, is widely used as a CYP2E1 probe for in vivo studies. 6-hydroxychlorzoxazone is the major metabolite of CZN which is mostly formed via CYP2E1 in both rats and humans [13].

Therefore, the objective of this study was to determine the pharmacokinetic changes of CZN after baicalin treatment and to explore the correlation between these changes and baicalin concentrations. Moreover the study identified the mechanisms underlying these alterations in the C_max_, V_d_, t_1/2_ by evaluating the effects of baicalin on CYP2E1 activity and CZN protein binding in vitro.

## Materials and Methods

### Ethics Statement

This study was carried out strictly accordance with the Guide for the Care and Use of Laboratory Animals. All the experimental procedures reported here were reviewed and approved by the Zhengzhou University Animal Care and Use Committee.

### Animals

Female Sprague–Dawley rats (180–220 g) were purchased from the Laboratory Animal Center of Henan province. These animals were housed in a temperature-controlled room with a 12 h light-dark cycle, with free access to the standard laboratory chow and water. The rats were fasted overnight before the pharmacokinetic experiments.

### Chemicals and Reagents

Baicalin (>98.5% purity) was kindly gifted by Henan Provincial Institute of Food and Drug Control. Chlorzoxazone (>99.5% purity) was purchased from the National Institute for the Control of Pharmaceutical and Biological Products (Beijing, China). 6-hydroxychlorzoxazone was purchased from Toronto Research Chemicals Inc. Methanol was HPLC grade and purchased from Siyou Chemical Reagent Co. (Tianjin, China). Reduced nicotinamide adenine dinucleotide phosphate (NADPH) was purchased from Solarbio Science and Technology co. Ltd (Beijing, China). All the other reagents were of analytical grade. Mill-Q water (Millipore, USA) was used throughout the study.

### Effects of Baicalin Treatment on CZN Pharmacokinetics

Eighteen female Sprague–Dawley rats were chosen to conduct this experiment. Animal experiments were designed to test the effect of co-administering CZN with baicalin on the pharmacokinetic profile of CZN in single dose as well as multidose pretreatment studies. The study was a randomized, three-period crossover design at intervals of 4 days. Drug dosing was done via the tail vein in all the pharmacokinetic studies.

#### Different doses of baicalin treatment

Nine rats were divided into three groups to receive an i.v. dose of saline (control) or 225 mg/kg baicalin or 450 mg/kg baicalin in the phase I. After the pretreatment an i.v. dose (15 mg/kg) of CZN was given immediately. The study was repeated twice with a washout period of 4 days. Blood samples were collected at predose, 0, 5, 15, 30, 60, 90 and 120 min by puncture of the orbital venous sinus. Plasma was separated from the blood by centrifugation at 4000 rpm for 10 min. The plasma samples were stored at −30°C until analysis.

#### Seven-day baicalin treatment

Nine rats were divided into two groups to receive a dose of saline (control) or 450 mg/kg baicalin in the phase I. After that CZN was given immediately. Then the study was repeated once again in the phase II. In the phase III, nine rats were pretreated with baicalin (450 mg/kg/d) for 7 days. During the pretreatment period, the rats were kept in a 12 h light–dark cycle animal room. Rats were allowed free access to diet and water. Immediately after the final pretreatment, the experiment was performed as described in the previous section.

Moreover pooled plasma obtained from rats (n = 9) treated by baicalin at 450 mg/kg in different doses of baicalin studies was used for CZN protein-binding analysis.

### Determination of Plasma CZN and Baicalin Concentration

The plasma concentration of CZN was determined according to the method of Chittur SV et al with modifications [14]. Briefly, the separation of CZN was achieved by using a Diamond C_18_ column (4.6×200 mm, 5 µm), with a mobile phase of methanol and water (60∶40, v/v) at a flow rate of 1 mL·min^−1^ and 25°C column temperature. The UV detection wavelength was 287 nm. The extraction method came from Rockich K and Blouin R with modifications [15]. Briefly, 1 ml acetic ether was added to 0.1 ml of plasma from each sample and vortexed for 2 min. The samples were centrifuged and the organic phase was evaporated to dryness under a stream of nitrogen. The residue was reconstituted in 100 µl of mobile phase and 40 µl was injected to the HPLC system.

The plasma concentration of baicalin was determined according to the method of Zeng MF et al with modifications [16]. An aliquot of 25 µl plasma was precipitated with 100 µl methanol, vortexed for 1 min and centrifuged for 10 min at 15000 rpm. 5 µl clear supernatant was injected to the HPLC system. The mobile phase consisted of methanol and 2% phosphoric acid (68∶32, v/v) at a flow rate of 1 mL·min^−1^ and 25°C column temperature. The UV detection wavelength was 278 nm.

### Measurement of Rat Plasma Protein Binding of CZN in vivo and in vitro [15]

The protein-binding of CZN in pooled plasma (n = 9) at different sampling times after treatment with baicalin (450 mg/kg, iv) were evaluated by ultrafiltration.

In vitro experiment, binding of CZN to protein in fresh plasma from female SD rats (*n* = 5) was measured. A 0.5 ml plasma sample was spiked to give a final CZN concentration of 50 mg·L^−1^. Plasma baicalin concentrations varied from 0 to 2500 mg·L^−1^ by adding of baicalin to plasma samples. The samples were incubated for 15 min at 37°C and 0.3 ml aliquots were placed into an ultrafiltration device (Millipore, USA). The samples were centrifuged at 3200 rpm for 25 min. Concentrations of CZN in the filtrate were determined by the HPLC.

### Effects of Baicalin on CYP2E1 Activity in vitro

The incubation mixture contained rat liver microsomes (0.375 mg/mL), NADPH (1 mM), 100 mM phosphate buffer (pH 7.4), CZN and baicalin at different concentrations. To determine the IC_50_ of baicalin, the concentration of CZN in incubation mixture was chosen to approach K_m_, and a series of baicalin were in the range 12.5∼400 µM. To estimate the Ki value, different concentrations of CZN (6.25∼200 µM) and baicalin (0, 50, 100, and 200 µM) were used.

After pre-incubating the mixture at 37°C for 5 min, the reaction was started by adding the NADPH and incubated for 30 min at 37°C. After the incubation, the reaction was stopped by placing into ice-bath. The method of determining 6-OH-chlorzoxazone was same as CZN. The kinetic constants (K_m_ and V_max_) for the formation of 6-OH-chlorzoxazone were calculated using the nonlinear regression method.

### Statistics

Pharmacokinetic analysis of data was calculated by DAS 2.0 (Mathematical Pharmacology Professional Committee of China, Shanghai, China). The results were reported as mean±SD. The maximum concentration (C_max_) and minimum concentration (C_min_) were determined by inspecting the individual drug plasma concentration–time profiles. Statistical analysis was performed with SPSS 17.0 software (SPSS Inc., Chicago, IL, USA). The significance of difference between groups was analyzed by paired sample *t* test. The level of significance was set at *P*<0.05.

## Results

### Effect of Baicalin Treatment on CZN Pharmacokinetics in Rats

#### Pharmacokinetics of baicalin

**Figure 1:**
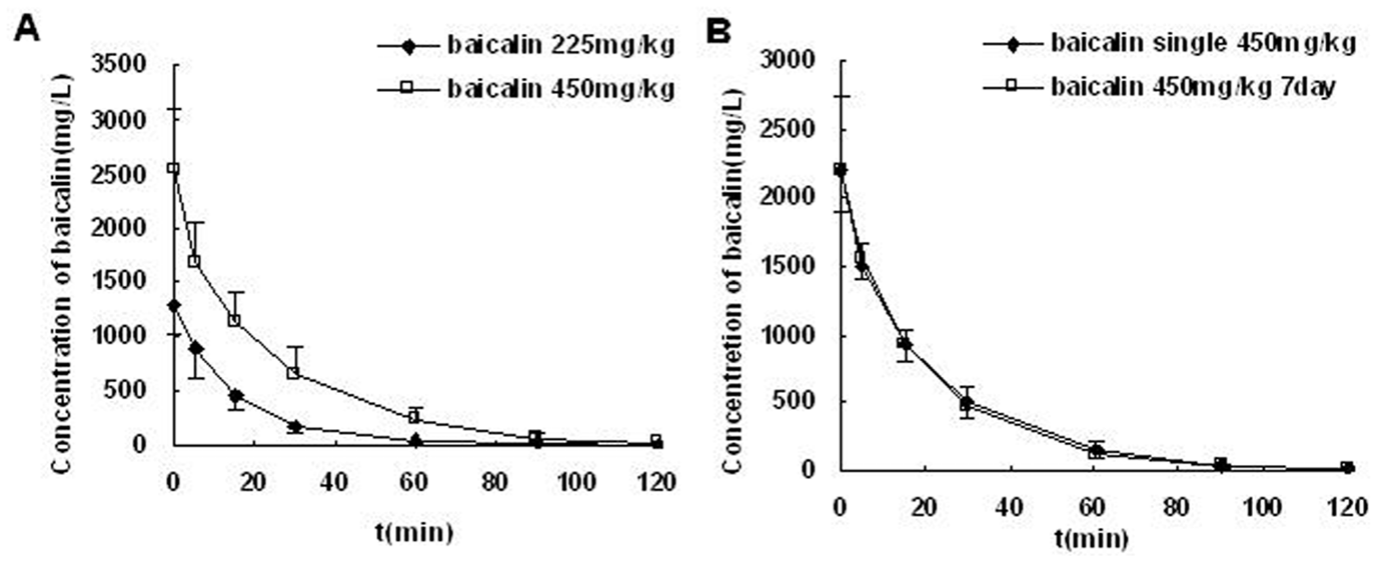
Mean plasma concentration–time profiles of baicalin in rats. (A) after i.v. administration baicalin at 225 mg/kg and 450 mg/kg. (B) after i.v. administration baicalin at 450 mg/kg/day for 1 day and 7 days in rats (mean ± SD, n = 9).

The baicalin plasma concentration–time curves were shown in Figure 1. The C_max_ in rats treated with baicalin at 225 mg/kg and 450 mg/kg were (1290±255) and (2543±564) mg/L respectively. Furthermore, the differences of main parameters between the rats after 1-day treatment and 7-day treatment with baicalin (450 mg/kg/day, i.v) were not significant (Data were not shown).

#### Different doses of baicalin treatment

**Figure 2:**
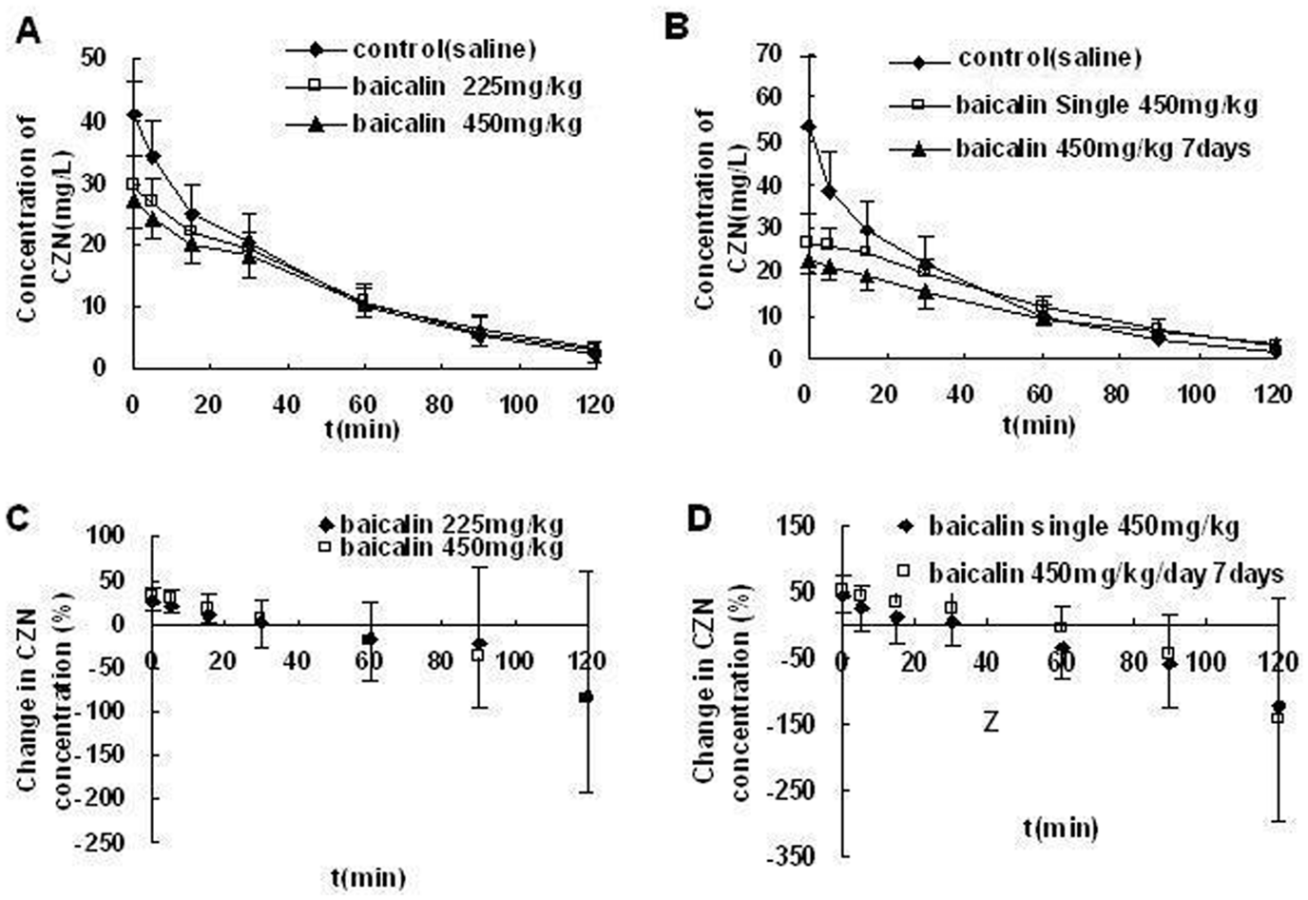
Effect of baicalin treatment on CZN pharmacokinetics. (A) (B)The concentration-time profile of CZN (15 mg/kg,iv) after treatment with saline (control) or baicalin in rats. (C) (D) The changes in CZN (15 mg/kg, i.v) concentrations (%) after treatment with baicalin compared with control. (A) (C) treatment with baicalin (225 or 450 mg/kg, i.v). (B) (D) 1 day-treatment or 7 day-treatment with baicalin (450 mg/kg/day, i.v). Each point represent the mean±SD (n = 9).

The plasma CZN concentration–time profiles after single pretreatment with baicalin (225 or 450 mg/kg, i.v) or saline (control) were shown in Figure 2A. This clearly illustrated that the administration of baicalin to female Sprague-Dawley rats significantly altered the pharmacokinetics of CZN. Table 1 listed the pertinent pharmacokinetic parameters for the control and treated groups. The C_max_ decreased by 25% and 33%, whereas t_1/2_ increased by 34% and 53%, V_d_ increased by 37% and 50% in different doses (225 and 450 mg/kg) of baicalin treated rats, respectively. No significant effects on the CL and AUC of CZN were observed by the single baicalin treatment. The changes in CZN concentrations (%) at different sampling times after treatment with baicalin (225 or 450 mg/kg, i.v) compared with saline control were shown in Figure 2C. It clearly illustrated that CZN concentration decreased at first and increased subsequently after treatment with baicalin.

**Table 1:** Pharmacokinetics of CZN (15 mg/kg,i.v) after treatment with different doses of baicalin (225 and 450 mg/kg,i.v).

	C_max_ (mg/L)				C_min_ (mg/L)			T_1/2_ (min)			Vd (L/kg)			CL (L/h/kg)			AUC (mg/L*min)		
	Control		Baicalin 225	Baicalin 450	Control	Baicalin 225	Baicalin 450	Control	Baicalin 225	Baicalin 450	Control	Baicalin 225	Baicalin 450	Control	Baicalin 225	Baicalin 450	Control	Baicalin 225	Baicalin 450
1	44.84		24.35	29.21	0.52	0.80	0.88	22.0	32.5	35.0	0.32	0.61	0.48	0.61	0.78	0.58	1421	1077	1495
2	40.58		26.83	29.32	1.62	3.43	0.74	28.0	45.1	38.3	0.36	0.56	0.50	0.54	0.52	0.55	1662	1705	1547
3	48.82		34.83	26.97	2.45	2.90	6.10	25.8	38.4	62.5	0.32	0.44	0.62	0.52	0.48	0.41	1842	1842	2166
4	48.42		31.72	19.13	2.79	1.50	5.53	32.5	35.2	56.5	0.31	0.51	0.73	0.40	0.60	0.53	2202	1430	1751
5	38.99		32.52	28.4	7.33	3.74	6.84	47.4	44.1	55.1	0.37	0.52	0.53	0.33	0.49	0.40	3023	1804	2305
6	34.46		32.23	31.23	1.06	1.39	2.31	22.5	34.9	32.7	0.44	0.50	0.51	0.81	0.60	0.64	1198	1457	1410
7	38.67		28.25	24.5	1.05	2.38	2.48	31.1	40.6	46.7	0.40	0.55	0.60	0.53	0.56	0.54	1656	1546	1762
8	36.07		28.38	23.32	2.52	4.44	2.10	37.2	43.7	46.3	0.45	0.55	0.64	0.51	0.53	0.58	1945	1743	1667
9	37.01		37.12	34.26	1.09	5.73	4.20	25.2	49.9	42.9	0.41	0.41	0.48	0.68	0.34	0.47	1356	2743	1928
x	40.87		30.69**	27.37**	2.27	2.92	3.46	30.2	40.5**	46.2**	0.38	0.52**	0.57**	0.55	0.54	0.52	1812	1705	1781
sd	5.29	4.06		4.52	2.06	1.59	2.28	8.1	5.7	10.2	0.05	0.06	0.08	0.14	0.12	0.08	550	456	303

** *vs* control *P*<0.01.

#### Seven-day baicalin treatment

Figure 2B showed the plasma CZN concentration–time profiles and Figure 2D showed the changes in CZN concentrations (%) compared with saline control after 7 day-treatment or 1 day-treatment with baicalin (450 mg/kg/day, i.v). From the pharmacokinetic parameters in Table 2, it could conclude that the C_max_ decreased while t_1/2_ and V_d_ increased in both of the treatment groups. Furthermore, in multiple pretreatment the effect of baicalin on C_max_ of CZN was observed to be significantly lower compared with the single dose administration (*P*<0.01).

**Table 2:** Pharmacokinetics of CZN (15 mg/kg,i.v) after a 1 day and 7 day treatment with baicalin (450 mg/kg,i.v).

	C_max_ (mg/L)			C_min_ (mg/L)			T_1/2_ (min)			Vd (L/kg)			CL (L/h/kg)			AUC (mg/L*min)		
	Control	Baicalin Single	Baicalin multiple	Control	Baicalin Single	Baicalin multiple	Control	Baicalin Single	Baicalin multiple	Control	Baicalin Single	Baicalin multiple	Control	Baicalin Single	Baicalin multiple	Control	Baicalin Single	Baicalin multiple
1	42.85	22.02	21.93	1.46	3.16	3.42	24.7	53.1	52.9	0.35	0.65	0.67	0.60	0.51	0.53	1547	1705	1639
2	72.44	26.97	25.43	0.84	5.06	4.50	17.8	53.7	54.0	0.22	0.56	0.60	0.53	0.43	0.46	1885	2046	1876
3	59.39	22.78	21.15	1.97	1.63	2.76	23.0	48.0	54.6	0.29	0.63	0.74	0.52	0.54	0.56	2027	1631	1584
4	70.16	21.94	19.12	3.46	1.32	3.17	23.9	35.1	46.6	0.24	0.64	0.78	0.41	0.76	0.70	2379	1126	1284
5	19.42	28.88	20.74	1.02	3.16	5.68	30.3	43.6	59.6	0.76	0.55	0.76	1.04	0.53	0.53	853	1645	1813
6	58.37	39.74	28.53	1.14	1.29	1.71	23.5	37.0	37.7	0.26	0.38	0.55	0.46	0.43	0.60	1963	2001	1455
7	56.49	27.82	22.72	2.16	2.59	1.75	25.1	56.7	32.4	0.28	0.61	0.65	0.46	0.45	0.84	1989	1895	1085
8	48.8	32.96	24.87	1.41	3.07	1.60	26.7	46.8	40.0	0.30	0.44	0.57	0.46	0.39	0.59	1934	2348	1571
9	54.04	28.86	25.34	2.08	6.61	5.92	21.8	57.6	51.8	0.27	0.57	0.57	0.52	0.41	0.46	1821	2227	2019
x	53.55	28.00**	23.31** ^ΔΔ^	1.73	3.10	3.39*	24.1	47.9**	47.7**	0.33	0.56**	0.65** ^ΔΔ^	0.56	0.49	0.58	1822	1847	1592
sd	15.81	5.75	2.94	0.80	1.77	1.66	3.4	8.2	9.1	0.17	0.09	0.09	0.19	0.11	0.12	423	369	293

* *vs* control *P*<0.05.

** *vs* control *P*<0.01.

ΔΔ*vs* single *P*<0.01.

### Correlation of CZN Changes and Baicalin Concentrations or Pharmacokinetic Parameters

#### Correlation between CZN concentration changes and baicalin

**Figure 3:**
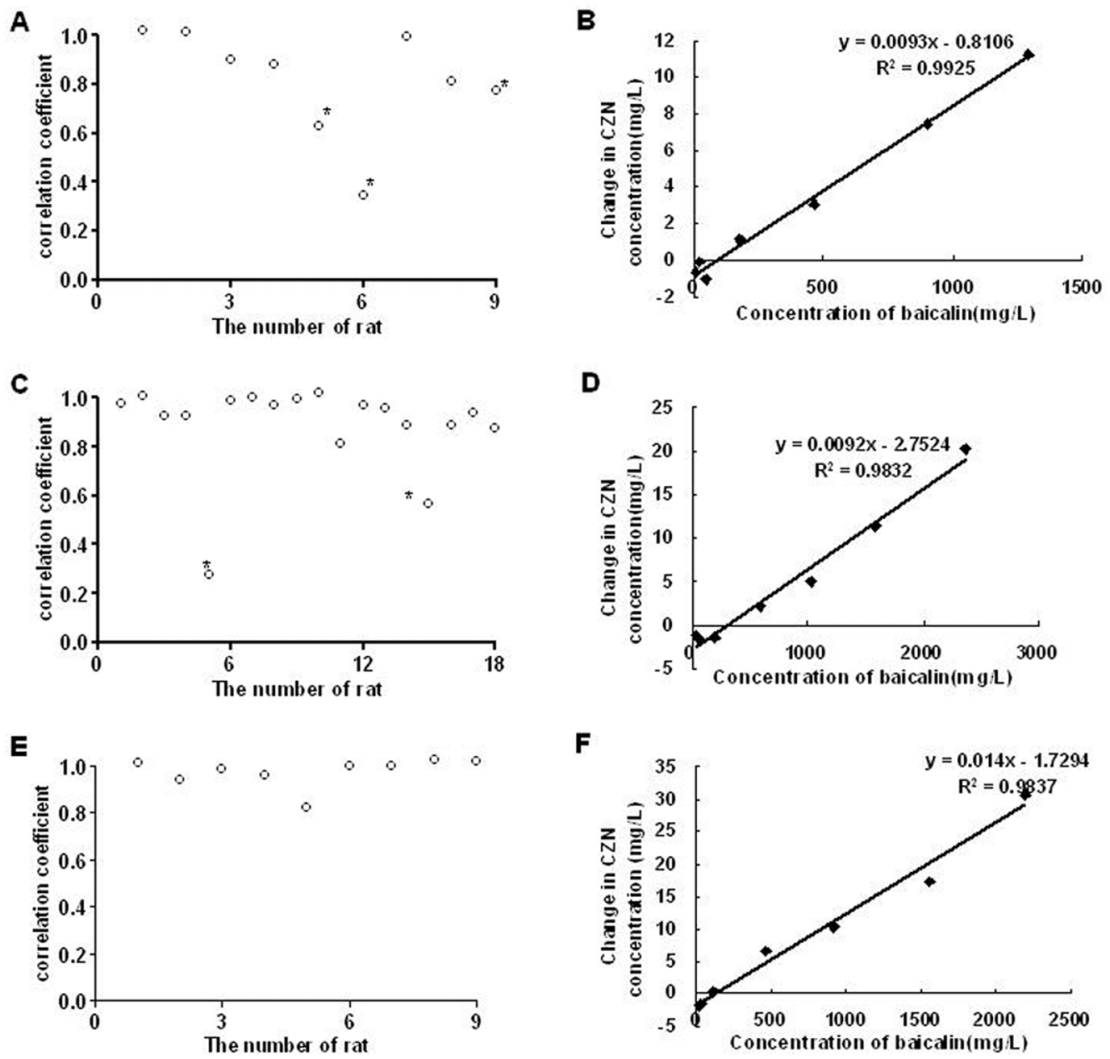
Correlation of changes in CZN concentrations and corresponding baicalin concentrations in rats. (A), (C), (E) Correlation coefficient of changes in CZN concentrations in different sampling times and corresponding baicalin concentrations in rats. (B), (D), (F) Plot of mean changes in CZN concentrations after baicalin treatment versus mean baicalin concentrations in rats. (A), (B) The rats treated with single dose of baicalin at 225 mg/kg (n = 9). (C), (D) The rats treated with single dose of baicalin at 450 mg/kg (n = 18, 9 rats were in different doses and 9 rats were in multiple dose). (E), (F) The rats treated with baicalin at 450 mg/kg/d for 7 days (n = 9). * *P*>0.05 in correlation analysis.

We explored the correlation between the changes in CZN concentrations in different sampling times and corresponding baicalin concentrations in rats after baicalin treatment (Figure 3A,C,E). The results showed that there were no significant correlations only in two rats treated with baicalin at 450 mg/kg and three rats treated with baicalin at 225 mg/kg (*P*>0.05). Figure 3B, D, F were correlation plots of the mean changes in CZN concentrations after baicalin treatment versus mean baicalin concentrations in rats. The correlation coefficients (*r* ^2^) were 0.9925, 0.9832 and 0.9837 in different pretreated rats with baicalin. It suggested a good prediction of baicalin effect on plasma concentration of CZN from the value of baicalin plasma concentration.

#### Correlation between CZN pharmacokinetic parameters changes and baicalin

**Figure 4:**
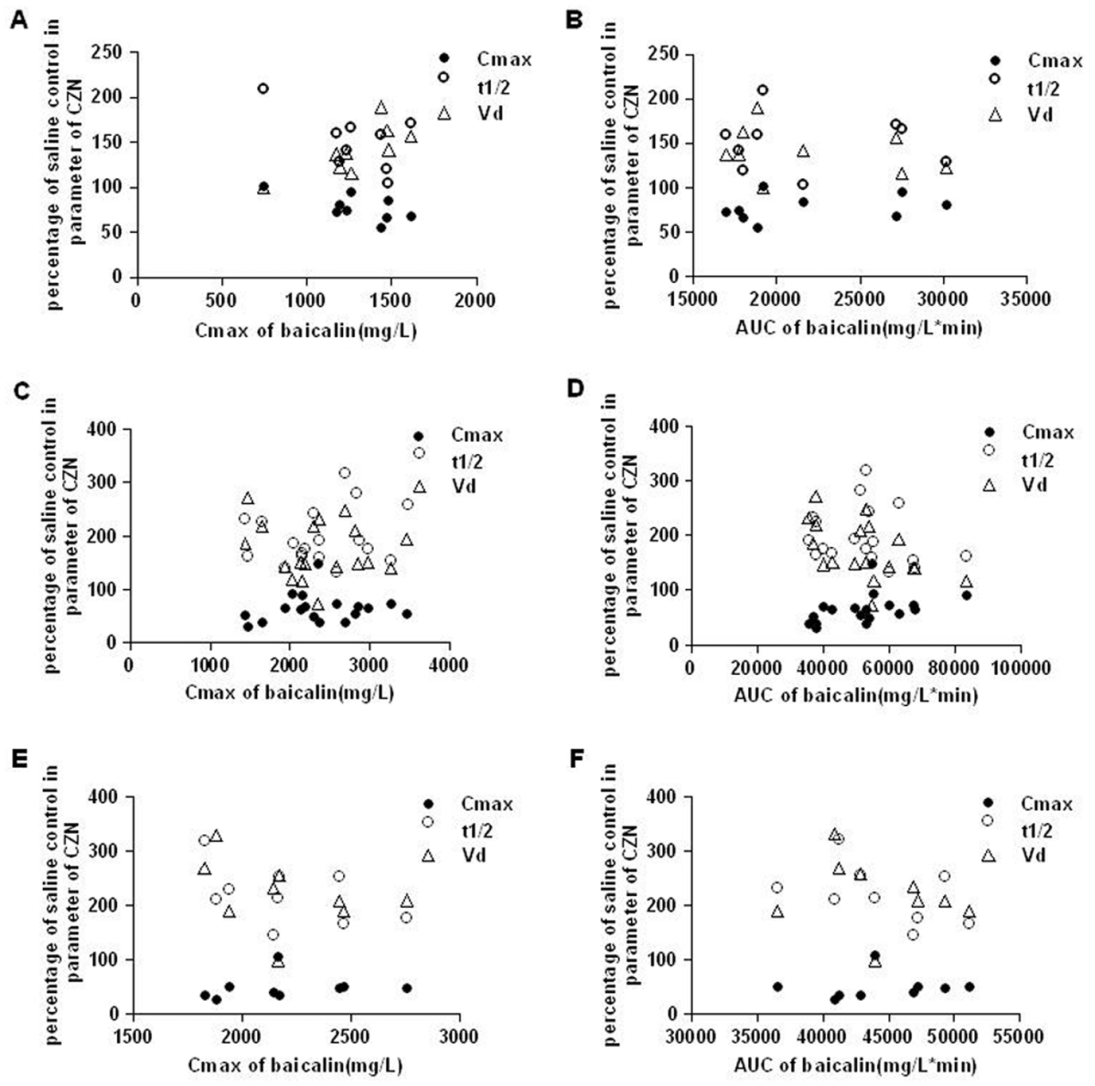
Plots of percentage of control in parameters of CZN versus C_max_ or AUC of baicalin. (A) (B) The rats treated with single dose of baicalin at 225 mg/kg (n = 9). (C) (D)The rats treated with single dose of baicalin at 450 mg/kg (n = 18, 9 rats were in different doses and 9 rats were in multiple dose). (E) (F) The rats treated with multiple dose of baicalin at 450 mg/kg (n = 9).

We studied the correlations between percentage of saline control in different parameters of CZN such as C_max_, t_1/2_, V_d_ and C_max_, AUC of baicalin after baicalin treatment. Excepted C_max_, V_d_ of CZN in rats treated with baicalin at 225 mg/kg and C_max_ of baicalin, there were no significant correlations (Figure 4).

### Plasma Protein Binding of CZN in vivo and in vitro

In order to explain why CZN concentration decreased at first after treatment with baicalin, we studied plasma protein binding of CZN in vivo and in vitro.

**Figure 5.The:**
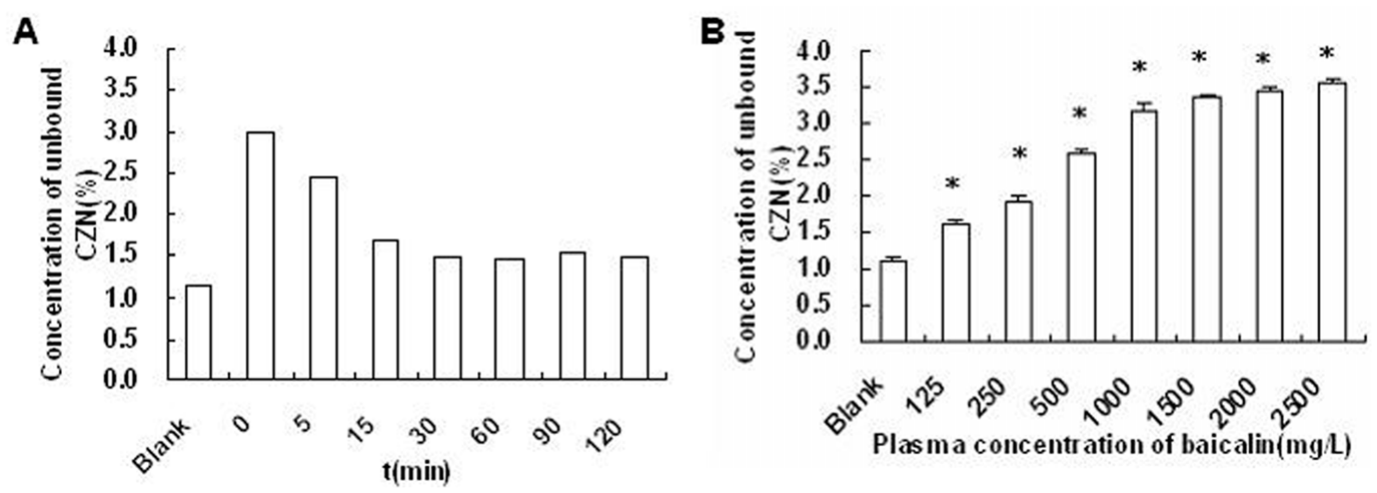
plasma protein binding of CZN. (A) The concentration of unbound CZN (%) in pooled plasma samples at different sampling times from rats after treatment with baicalin (450 mg/kg, iv, n = 9) (B) Effect of baicalin in concentration from 125–2500 mg/L on concentration of unbound CZN (%) in pooled rat plasma (n = 5). Total CZN concentration was 50 mg/L. * indicates a significant (*P*<0.05) increase in concentration of unbound CZN (%) from blank values.

Figure 5A listed the concentration of unbound CZN (%) in pooled plasma samples at different sampling times from rats (n = 9) after treatment with baicalin (450 mg/kg, iv). Our results showed that the concentration of unbound CZN was 1.14% without baicalin in vitro, while the unbound CZN increased by 163%, 115% and 50% at 0, 5, 15 min, and increased by about 30% from 30 min to 120 min compared with control (1.14%) in vivo.

As shown in Figure 5B, when the concentration of baicalin was 1000 mg·L^−1^, which was equivalent to the C_max_ value in rat treated with baicalin at 225 mg/kg, the concentration of unbound CZN (%) increased approximately 3-fold. Baicalin at different concentrations (125, 250, 500, 1000 and 1500 mg·L^−1^) increased the concentrations of unbound CZN (%) linearly (y = 0.0013x+1.69, *R* = 0.95) from 1.63% to 3.37%. Meanwhile we found that the concentrations of unbound CZN increased from 3.37% to 3.58% when concentrations of baicalin increased from 1500 mg·L^−1^ to 2500 mg·L^−1^. The results indicated that the concentration of unbound CZN (%) increased caused by the enhanced concentration of baicalin and had a steady trend.

### Effects of Baicalin on CYP2E1 Activity in vitro

**Figure 6:**
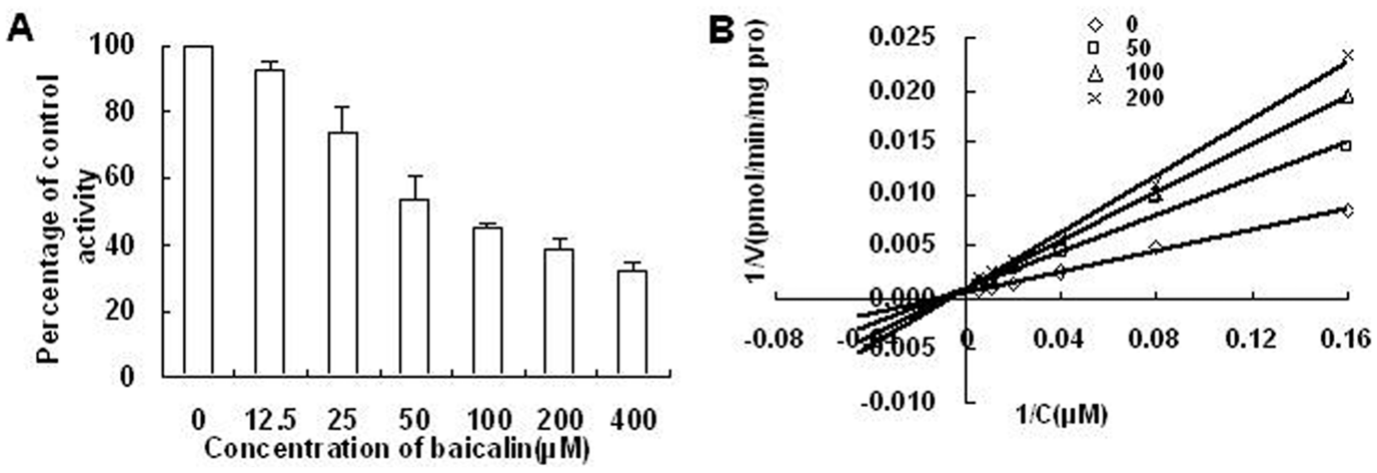
Inhibition of CYP2E1 activities by baicalin. (A) Inhibition of CYP2E1 activities by baicalin in pooled rat liver microsomes, presented as percentage of control activities (the concentration of CZN is 50 µM). (B) Lineweaver-Burk plots of the effect of baicalin on 6-hydroxychlorzoxazone formation in rat liver microsomes. Reactions were performed in the presence of CZN (6.25, 12.5, 25, 50, 100, 200 µM) and various concentrations of baicalin (0, 50, 100, 200µM) in the microsome (0.375 mg/mL) and NADPH-generating system in 100 mM phosphate buffer (pH 7.4), in a final volume of 200µL at 37°C for 30 min. All the data presented are from an analysis of the means of three separate experiments.

Analysis of enzyme kinetics indicated that the K_m_ and V_max_ values for CZN in rat liver microsomes were 89.7 µM, and 1892 pmol/min/mg protein, respectively. To investigate whether baicalin affected the activity of CYP2E1 in rats, the probe reaction assays were conducted with various concentrations of baicalin. Results showed that baicalin acted as a competitive inhibitor of rat CYP2E1, which is responsible for the metabolism of CZN to 6-hydroxychlorzoxazone, with IC_50_ and Ki values (Figure 6) of 103.5 µM and 145.8 µM respectively.

## Discussion

Inhibition of CYP-dependent metabolism is one of the most common mechanisms leading to drug–drug interactions and results in decreased drug clearance and drug accumulation in target cells, which may lead to serious clinical consequences. Assessment of the potential for herb to cause herb–drug interactions via inhibition of CYP-dependent metabolism is important in the drug discovery process. Although some works on the effect of baicalin to CYP have been investigated, many questions about the action of baicalin to CYP are still unclear because of inappropriate route and dose of administration.

To clarify the inhibitory effect of baicalin on CYP2E1 mediated metabolism, single pretreatment studies at two doses were scheduled using CZN as a probe drug. Furthermore we conducted the multiple-pretreatment study. The rats in our experiment were female. It was reported that differences in the levels of CYP2E1 expression between male and female rats indicated the hormonal control of CYP expression and CYP2E1 is dominant in female rat liver [17], [18]. Rockich K et al had explored CZN pharmacokinetics after i.v CZN (15 mg/kg) in male rat [15]. The main pharmacokinetics parameters such as t_1/2_ and V_d_ were (30.5±1.78) min and (0.39±0.04) L/kg, respectively and were similar to our results. This suggested that the gender of rat had little effect on the metabolism of CZN by CYP2E1.

The results showed that the C_max_ decreased after treatment with baicalin and both V_d_ and t_1/2_ increased not only in single dose group but also in multiple dose group. Plasma protein binding plays an important role in the whole-body disposition of drugs and it should be taken into account when interpreting changes in total plasma concentrations of drugs. It was reported that the plasma protein binding of baicalin was in the range of 86%–92% [19]. Meanwhile CZN is a low extraction drug, it is important to measure the change in the protein binding of CZN [15]. In this study, the free-fraction in the rat treated with baicalin increased compared with control values. Our results showed that baicalin had inhibition on CYP2E1 of rat in vitro, but the CL in vivo was not change after administration baicalin. The reason may be as follows. The increase in free fraction of a drug by a displacer will increase its hepatic clearance and may cover a concomitant effect of the displacer as an enzyme inhibitor in decreasing drug clearance [20]. The intrinsic clearance (CL_int_) is calculated from CL/fu, where fu is the free-fraction of CZN in the plasma [15]. Although there was no difference in CL, intrinsic clearance decreased owing to the increase of unbound drug. The Cl_int_ is defined as the enzyme’s inherent ability to remove drug from the body [21]. These results also may explain why no change in the CL whereas inhibition of baicalin on CYP2E1 in vitro was observed.

V_d_ increased by 37% and 50% in different doses (225 and 450 mg/kg) of baicalin treated rats compared with controls in the study, respectively. This may be explained by the increase of the unbound-fraction of CZN in the baicalin treatment group. A decrease in drug plasma protein binding in vivo may decrease the total plasma drug level because of redistribution of unbound drug to extravascular sites, and/or an increase in clearance. On the other hand, when the concentration of baicalin increased from 1000 mg·L^−1^ to 2500 mg·L^−1^ (2.5 fold), the concentration of unbound CZN only increased from 3.19% to 3.58% (1.12 fold). While the C_max_ values of baicalin in rats treated with baicalin at 225 mg/kg and 450 mg/kg were 1290 mg/L and 2543 mg/L, respectively. The results may partly explain why there was no difference in C_max_ values of CZN between rats treated with baicalin of 225 mg/kg and 450 mg/kg.

To obtain further information on the effect of baicalin on the activity of CYP2E1, an in vitro study was also carried out. The K_m_ and V_max_ values of the CZN obtained from the experiments were close to the values reported in the literature [22].The present in vitro study showed that baicalin acts as a competitive inhibitor of rat CYP2E1, which is responsible for the metabolism of CZN to 6-OH-chlorzoxazone, with IC_50_ and Ki values of 103.5 µM and 145.8 µM, respectively. Given the relatively large Ki values, it seemed that baicalin should not pose a major problem in herb–drug interaction with CYP2E1 substrates. But our results showed that the C_max_ values of baicalin in rat treated with baicalin at 225 mg/kg and 450 mg/kg were 1290 mg·L^−1^ and 2543 mg·L^−1^, which were equivalent to 2890 µM and 5697 µM. So such metabolic interaction had been confirmed in vivo experiment. Correlation analysis showed that the changes in CZN concentration and concentrations of baicalin were in good correlation. It meant that we could predict the effect of baicalin on plasma concentration of CZN from the data of baicalin plasma concentration.

In this study, self-control was used to observe the effect of the baicalin on CZN pharmacokinetics. With this approach, we could find the individual difference. For example, there was no significant correlation in the 5th rat treated by baicalin at 225 mg/kg (Figure 3A). The reasons for individual difference remain a problem and need study further.

In summary, the study demonstrated that baicalin administration produced significant increase in V_d_ of CZN and decrease in C_max_ secondary to reductions in its plasma protein binding. Though there was no difference in CL, CL_int_ decreased owing to increase of unbound drug and these changes were in accordance with inhibition of CYP2E1 activity in vitro.
